# Effects of sheared chromatin length on ChIP-seq quality and sensitivity

**DOI:** 10.1093/g3journal/jkab101

**Published:** 2021-03-31

**Authors:** Cheryl A Keller, Alexander Q Wixom, Elisabeth F Heuston, Belinda Giardine, Chris C -S Hsiung, Maria R Long, Amber Miller, Stacie M Anderson, April Cockburn, Gerd A Blobel, David M Bodine, Ross C Hardison

**Affiliations:** 1 Department of Biochemistry and Molecular Biology, The Pennsylvania State University, University Park, PA 16802, USA; 2 Mayo Clinic, Department of Gastroenterology and Hepatology, Rochester, MN 55905, USA; 3 NHGRI Hematopoiesis Section, Genetics and Molecular Biology Branch, National Institutes of Health, Bethesda, MD 20892, USA; 4 Department of Pathology, Stanford University School of Medicine, Stanford, CA 94305, USA; 5 Department of Urology, University of California, San Francisco, San Francisco, CA 94158, USA; 6 NHGRI Flow Cytometry Core, National Institutes of Health, Bethesda, MD 20882, USA; 7 Division of Hematology, The Children's Hospital of Philadelphia, Philadelphia, PA 19104, USA; 8 Perelman School of Medicine, University of Pennsylvania, Philadelphia, PA 19104, USA

**Keywords:** ChIP-seq, chromatin immunoprecipitation, reproducibility, sonication, genomics, hematopoiesis, hematopoietic progenitors, CTCF, TAL1

## Abstract

Chromatin immunoprecipitation followed by massively parallel, high throughput sequencing (ChIP-seq) is the method of choice for genome-wide identification of DNA segments bound by specific transcription factors or in chromatin with particular histone modifications. However, the quality of ChIP-seq datasets varies widely, with a substantial fraction being of intermediate to poor quality. Thus, it is important to discern and control the factors that contribute to variation in ChIP-seq. In this study, we focused on sonication, a user-controlled variable, to produce sheared chromatin. We systematically varied the amount of shearing of fixed chromatin from a mouse erythroid cell line, carefully measuring the distribution of resultant fragment lengths prior to ChIP-seq. This systematic study was complemented with a retrospective analysis of additional experiments. We found that the level of sonication had a pronounced impact on the quality of ChIP-seq signals. Over-sonication consistently reduced quality, while the impact of under-sonication differed among transcription factors, with no impact on sites bound by CTCF but frequently leading to the loss of sites occupied by TAL1 or bound by POL2. The bound sites not observed in low-quality datasets were inferred to be a mix of both direct and indirect binding. We leveraged these findings to produce a set of CTCF ChIP-seq datasets in rare, primary hematopoietic progenitor cells. Our observation that the amount of chromatin sonication is a key variable in success of ChIP-seq experiments indicates that monitoring the level of sonication can improve ChIP-seq quality and reproducibility and facilitate ChIP-seq in rare cell types.

## Introduction

Chromatin immunoprecipitation followed by massively parallel, high throughput sequencing (ChIP-seq) has been used extensively to produce thousands of genome-wide maps of DNA segments bound by specific transcription factors (TFs) or in chromatin with particular histone modifications. However, these ChIP-seq datasets vary widely in quality. A uniform analysis of vertebrate TF ChIP-seq datasets in the Gene Expression Omnibus (GEO) repository (RRID: SCR_005012) found that a substantial fraction were of intermediate to poor quality, and many of the control datasets (*e.g.*, IgG and mock immunoprecipitations) displayed enrichments similar to the experimental datasets, indicating that many ChIP-seq results are not substantially different from the negative controls (Marinov *et al.* 2014). An independent assessment of reproducibility in ENCODE ChIP-seq datasets found that while almost half the datasets had good agreement in peak calls across replicates, almost one-third (18/57) were dissimilar between replicates (Devailly *et al.* 2015). The variation in quality of ChIP-seq datasets is well-known, and efforts have been made to communicate the quality to potential users. A set of quality metrics were defined (Landt *et al.* 2012), and these are used to evaluate datasets within ENCODE and frequently in individual labs. Application of these and additional considerations has led to the introduction of audit “flags” at the ENCODE data portal (RRID: SCR_015482; Sloan *et al.* 2016; Davis *et al.* 2018). For the 7547 ChIP-seq datasets across four species in ENCODE (as of July 14, 2020), about 300 red flags and about 3000 orange flags were given (multiple flags can be assigned to one dataset), illustrating a serious, but not crippling, issue. The variable quality in ChIP-seq datasets, documented in these studies, leads to increased costs due to failed experiments, and it can lead to misinterpretation of data when the failures or very low quality of datasets are not recognized. Thus, it is important to discern and control the factors that contribute to variation in ChIP-seq.

Some of the factors that affect quality and reproducibility of ChIP-seq data are largely outside the control of the experimentalist. The major limiting factor in conventional ChIP-seq is the availability of highly specific antibodies. While many manufacturers make claims of “ChIP-seq validated” antibodies, many antibodies do not produce high-quality ChIP-seq data, resulting in a high failure rate using commercially available antibodies against a diverse spectrum of TFs (Savic *et al.* 2015).

Additional variables that influence the success of a ChIP-seq experiment include the abundance of the target proteins and their accessibility in chromatin to antibodies. Assays of modified histones in chromatin are often highly sensitive and reproducible, likely due to the availability of good quality antibodies as well as the abundance of histones and their tight association with chromatin. By comparison, the abundance of sequence-specific TFs varies among factors as well as across cell types. Furthermore, the extent of interaction with chromatin varies depending on its binding site and whether or not it binds DNA directly or via other DNA-associated factors.

Variables such as antibody quality or antigen abundance and accessibility can sometimes be controlled, but only by dedicated effort focused on the TF or epigenetic feature of interest. Other variables that are part of the preparative procedure, such as specific chromatin fixation and sonication conditions, are controlled by the experimentalist, and they can be optimized to improve sensitivity and reproducibility of chromatin immunoprecipitation (Khoja *et al.* 2019). Previous studies of chromatin size in ChIP-seq have indicated a recommended size range between 100 and 300 bp (Landt *et al.* 2012; Browne *et al.* 2014), while other work suggests a wider size range of 100 to 600 bp (Diagenode 2012). Thus, the optimal size for ChIP-seq currently is not clear, and a convenient means for achieving an optimal size range has not been described.

In this study, we evaluated how the extent of chromatin sonication affects ChIP-seq quality and success rate for two different sequence-specific TFs, CTCF and TAL1, and for POL2, in mouse erythroid cells. We further develop a case that the distribution of sizes of chromatin fragments after sonication is a critical and controllable aspect of ChIP-seq experiments. Finally, we leveraged these findings to produce a set of CTCF ChIP-seq datasets in primary hematopoietic progenitor cells, including several rare cell types such as hematopoietic stem and progenitor cells and lineage-restricted progenitor populations.

## Materials and methods

### Cell culture methods

G1E-ER4 cells were grown in IMDM media + 15% FBS, kit ligand, and erythropoietin in a standard tissue culture incubator at 37°C with 5% CO^2^ as described (Weiss *et al.* 1997). To induce erythroid maturation, G1E-ER4 cells were treated with 10^−8 ^mol/L β-estradiol for 24 hours (G1E-ER4+E2). Cells were harvested by centrifugation at 500 × *g* for 5 minutes at 4°C and washed once in 1×PBS.

### Isolation of hematopoietic progenitor cells

All primary hematopoietic cell populations were enriched from 5- to 8-weeks-old C57BL6 male mice. LSK, CMP, MEP, GMP, CFU-E, ERY, CFU-MK, and MK populations were harvested and isolated from bone marrow (BM) using the following cell isolation markers as described (Heuston *et al.* 2018, Supplementary Table S4).

LSK: CD4^−^, CD8^−^, IL7Ra^−^, CD11b^−^, Ly6g/c^−^, CD45R^−^, Ter119^−^, cKit^+^, Sca1^+^

CMP: CD4^−^, CD8^−^, IL7Ra^−^, CD11b^−^, Ly6g/c^−^, CD45R^−^, Ter119^−^, cKit^+^, Sca1^−^, CD34^+^, CD16/32^−^

GMP: CD4^−^, CD8^−^, IL7Ra^−^, CD11b^−^, Ly6g/c^−^, CD45R^−^, Ter119^−^, cKit^+^, Sca1^−^, CD34^+^, CD16/32^+^

MEP: CD4^−^, CD8^−^, IL7Ra^−^, CD11b^−^, Ly6g/c^−^, CD45R^−^, Ter119^−^, cKit^+^, Sca1^−^, CD34^−^, CD16/32^−^

CFUE: CD4^−^, CD8^−^, IL7Ra^−^, CD11b^−^, Ly6g/c^−^, CD45R^−^, Ter119^+^, CD44^+^, FSCHigh

ERY: CD4^−^, CD8^−^, IL7Ra^−^, CD11b^−^, Ly6g/c^−^, CD45R^−^, Ter119^+^, CD44^+^, FSCLow

CFUMk: CD4^−^, CD8^−^, IL7Ra^−^, CD11b^−^, Ly6g/c^−^, CD45R^−^, Ter119^−^, Sca1^−^, CD41^+^, CD61^+^, cKit^+^

MK: CD4^−^, CD8^−^, IL7Ra^−^, CD11b^−^, Ly6g/c^−^, CD45R^−^, Ter119^−^, Sca1^−^, CD41^+^, CD61^+^, cKit^−^

### Chromatin immunoprecipitation (ChIP)

For CTCF or TAL1 in G1E-ER4+E2 cells, either 1, 5, 20, or 50 M cells in 1×PBS were crosslinked for 10 minutes by adding formaldehyde at a final concentration of 0.4%, and glycine was added at a final concentration of 125 mM to quench cross-linking. Cells were washed in 1×PBS and stored at −80°C until needed. For POL2 datasets, in either G1E-ER4 (YFP-MD) or G1E-ER4+E2 (YFP-MD) cells, chromatin immunoprecipitation was performed as described (Hsiung *et al.* 2016).

For CTCF in LSK, CMP, MEP, GMP, CFUE, ERY, CFUMk, MK, cells were fixed in 0.4% formaldehyde (16% methanol-free, Thermo Scientific) for 15 minutes before quenching in 125 mM glycine for 5 minutes. Cells were washed in 2× PIC (Roche mini-tabs, 1 tab in 5 ml = 2×) and stored at −80°C until needed.

Cells were then lysed (10 mM Tris-HCl, pH 8.0, 10 nM NaCl, 0.2% NP40) for 10 minutes on ice, washed once in 1× PBS, followed by nuclear lysis (50 mM Tris-HCl 8.0, 10 mM EDTA, 1% SDS) for 10 minutes on ice. Chromatin was then diluted further with Immunoprecipitation Buffer (20 mM Tris-HCl, pH 8.0, 2 mM EDTA, 150 mM NaCl, 1% Triton X-100, 0.01% SDS) and a 1× Protease Inhibitor Cocktail set V, EDTA-free (Calibiochem, La Jolla, CA, USA). A Diagenode Bioruptor Plus 300 (Diagenode Cat# B0102001) was used to shear samples in cycles of 30 seconds on, 30 seconds off sonication at high output power at 4°C for the desired number of cycles. For optimization of sonication, see the protocol detailed in Supplementary Figure S1.

Sonicated chromatin was pre-cleared overnight at 4°C with 8 μg of normal goat IgG (Santa Cruz Biotechnology, Santa Cruz, CA; sc2028) for TAL1 or 8 μg of normal rabbit IgG (sc2027) for CTCF. Separately, 5 μg of TAL1 antibody (Santa Cruz Biotechnology, sc12984x, lot B2511) or 5 μl of CTCF antiserum (MilliporeSigma, 07-729) were pre-bound to protein G agarose beads overnight at 4°C. For binding, pre-cleared chromatin was added to the antibody: bead complex and incubated with rotation at 4°C for 4 hours. Two hundred microliters of pre-cleared chromatin was saved for use as input. After binding, the beads were washed with Wash Buffer I (20 mM Tris-HCl, pH 8.0, 2 mM EDTA, 50 mM NaCl, 1% Triton X-100, 0.1% SDS), High Salt Wash Buffer (20 mM Tris-HCl, pH 8.0, 2 mM EDTA, 500 mM NaCl, 1% Triton X-100, 0.1% SDS), Wash Buffer II (10 mM Tris-HCl, pH 8.0, 1 mM EDTA, 250 mM LiCl, 1% NP-40, 1% deoxycholate), and 1×TE. DNA: protein complexes were then eluted from beads with Elution Buffer (1% SDS, 100 mM NaHCO_3_). Reverse crosslinking of immunoprecipitated chromatin was accomplished by the addition of NaCl to ChIP and input samples, followed by incubation overnight at 65°C with 1 μg RNase A. To remove proteins, each sample was treated with 6 μg Proteinase K for 2 hours at 45°C. Finally, immunoprecipitated DNA was then purified using the Qiagen PCR Purification Kit (Qiagen, Germantown, MD, USA).

### Illumina library preparation for ChIP-Seq

All samples, including inputs, were processed for library construction for Illumina sequencing using Illumina’s TruSeq ChIP-seq Sample Preparation Kit according to manufacturer’s instructions. The DNA libraries were sequenced on the HiSeq 2000 or NextSeq 500, as indicated (Supplementary Tables S1 and S3), using Illumina’s kits and reagents as appropriate.

### ChIP-seq data processing

The sequencing reads were mapped to mouse genome assembly mm10 (CTCF and TAL1) or mm9 (POL2) using Bowtie (0.12.8), and then filtered to remove chrM, unplaced chromosomes, and unmapped reads. The alignment was converted to bam format using Samtools 0.1.8., and MACS (1.3.7.1) was used to generate the wiggle tracks and call peaks. UCSC's wigToBigWig program was used to convert the wiggle file to a bigWig.

### Motif analysis of optimal peaks

Optimal IDR thresholded (high confidence) peak bed files were downloaded from the ENCODE portal (RRID: SCR_015482) (Sloan *et al.* 2016; Davis *et al.* 2018) for TAL1 (https://www.encodeproject.org/files/ENCFF972VNL/; last accessed April 6, 2021) and CTCF (https://www.encodeproject.org/files/ENCFF929RJU/; last accessed April 6, 2021). All peak bed files (both high confidence and our datasets) were converted to fasta files using bedtools (2.27.1) getfasta command. Each peak in the fasta files were screened for CTCF and TAL1 motifs (HOCOMOCOv11) (RRID: SCR_005409) using FIMO, from the MEME suite (5.2.0) (RRID: SCR_001783), to predict peaks with that motif (*P* < 0.0001). A combination of custom bash scripts and the bedtools intersect command was used to extract and quantitate peaks with and without motifs (Supplemental File S2). Visualization of the bigWigs of the retrospective datasets over optimal peaks regions was done using deepTools (3.5.0) (RRID: SCR_016366) computeMatrix, bamCoverage, plotHeatmap, and plotProfile.

### Selection criteria for retrospective datasets

The inclusion criteria for CTCF and TAL1 datasets were as follows: (1) ChIP-seq experiments were performed in the same mouse erythroid cell line, G1E-ER4+E2, (2) the factor immunoprecipitated was either CTCF or TAL1, and (3) we had available unenriched input chromatin corresponding to the ChIP-seq library so that we could measure the average length of the chromatin used for the corresponding ChIP. A total of 17 retrospective datasets (eight CTCF, nine TAL1) with independent chromatin sonications met these criteria. These ChIP-seq experiments were conducted over a variety of conditions, including different fixation methods. For POL2, we used a series of 25 published (Hsiung *et al.* 2016) and unpublished POL2 ChIP-seq datasets that includes three to four replicates per condition. Because these POL2 ChIP-seq experiments were part of a time-course experiment in which G1E-ER4+E2 cells were arrested in prometaphase by nocodazole treatment, followed by release into nocodazole-free medium for 40–360 minutes (Hsiung *et al.* 2016), and thus display little to no POL2 binding at the early time points, we excluded datasets that were less than 60 minutes after nocodazole release, and only compared replicates within each time point for our subjective assessment of dataset quality. Metadata for all retrospective datasets can be found in Supplementary Table S1.

## Data availability

**Figure 1:**
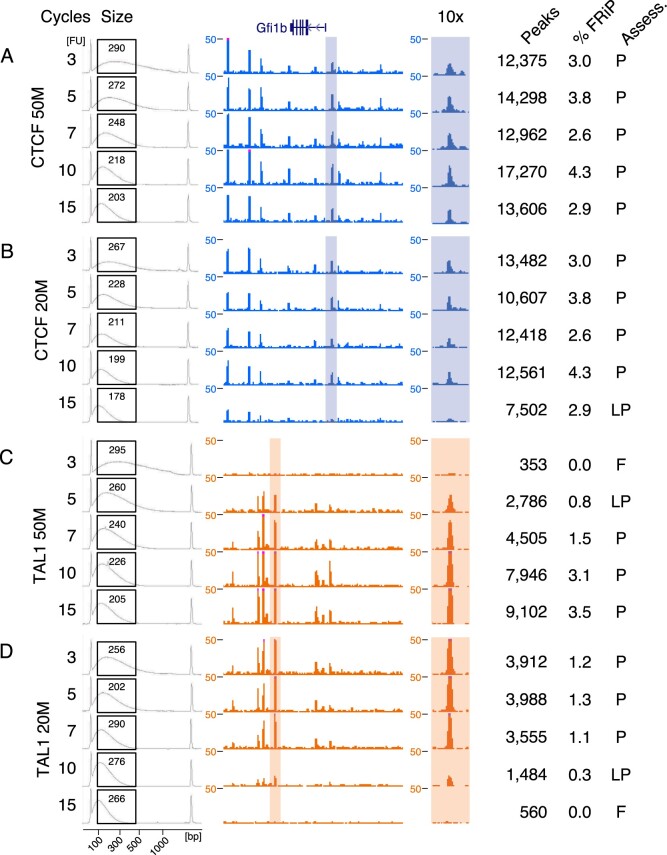
Bioanalyzer size distribution profiles and ChIP-seq signal tracks at the *Gfi1b* locus (chr2: 28,581,747-28,660,175). Bioanalzyer tracings (gray) show the size distribution profiles of the unenriched chromatin length. Black boxes overlaying the tracings denote the 100–500 bp region used to measure the average chromatin length (number in the box). Shaded areas in the signal tracks are also shown at 10× magnification to the right of the signal track, followed by the number of peaks observed genome-wide and the Fraction of Reads in Peaks as a percentage (% FRiP). The assessment (Assess.) is P, pass; LP, low pass; F, fail. (A) CTCF 50 M cells, (B) CTCF 20 M cells, (C) TAL1 50 M cells, and (D) TAL1 20 M cells.

Supplemental File S1 contains detailed descriptions of all supplemental files. Supplemental File S2 contains code used to analyze peaks and motifs. Supplementary Table S1 contains a list of datasets and metadata for all ChIP-seq samples. Supplementary Table S2 contains data on peaks and motifs statistics. Supplementary Table S3 contains a list of datasets and accompanying metadata for all input samples. Supplementary Table S4 contains isolation/sort markers, dates of collection, and cell numbers for hematopoietic progenitors. Supplemental Material available at figshare: https://doi.org/10.25387/g3.14237849. Data are deposited in the NCBI Gene Expression Omnibus (GEO; https://www.ncbi.nlm.nih.gov/geo/; last accessed April 6, 2021; RRID: SCR_005012), GEO accession number GSE159503. A custom UCSC genome session for the datasets in Figures 1 and 7 can be viewed at: https://main.genome-browser.bx.psu.edu/cgi-bin/hgTracks?hgS_doOtherUser=submit&hgS_otherUserName=cak142&hgS_otherUserSessionName=Keller_sonication_mm10; last accessed April 6, 2021.

## Results

To evaluate systematically how chromatin sonication affects ChIP-seq quality and success rate, we used the Diagenode Biorupter 300 to shear fixed chromatin from batches of 50 and 20 M mouse erythroid G1E-ER4+E2 cells to varying degrees (Supplementary Figure S1), assayed the extent of shearing using the Agilent Bioanalzyer 2100, and then subjected the chromatin to immunoprecipitation and sequencing using antibodies against either CTCF or TAL1 (Figure 1, Supplementary Table S1), for which binding sites are well known.

The resulting ChIP-seq patterns revealed a striking dependence on numbers of sonication cycles. While many CTCF ChIP-seq samples (Figure 1, A and B) showed the expected peaks at the illustrative locus *Gfi1b*, the sample from 20 M cells sonicated for 15 cycles showed low signal-to-noise (Figure 1B). Good quality TAL1 ChIP-seq data were obtained for most of the samples, but the pattern of failures was more complex, with poor results obtained at low cycle numbers for 50 M cells and at higher cycle numbers for 20 M cells (Figure 1, C and D).

We hypothesized that the lower quality datasets may result from the impact of the different cell numbers and sonication conditions on the resulting sizes of the chromatin. To investigate the relationship between sheared chromatin length and ChIP-seq success, we used the Bioanalyzer results to determine the average size of unenriched chromatin between 100 and 500 bp for each sample (Figure 1, Supplementary Table S1). This range was selected in order to standardize the measurement and avoid skewing the average by including large molecular weight heterochromatin. Further, fragments outside of this size range are less likely to be sequenced when using the Illumina platform. We stress that the average chromatin fragment sizes measured in this manner are not the same as the average library size of the completed library or the library insert sizes deduced from the patterns of mapped reads after sequencing. Those library sizes reflect the distribution of DNA fragments that were selected during the library preparation protocol; they are not the average size of the pool of unenriched chromatin. In other words, the DNA fragments measured by sequencing library size are the subset of immunoprecipitated chromatin that are most favorable for sequencing, whereas the measurements used in our study are those of the unenriched chromatin input for immunoprecipitation, which are controlled by the experimentalist.

**Figure 2:**
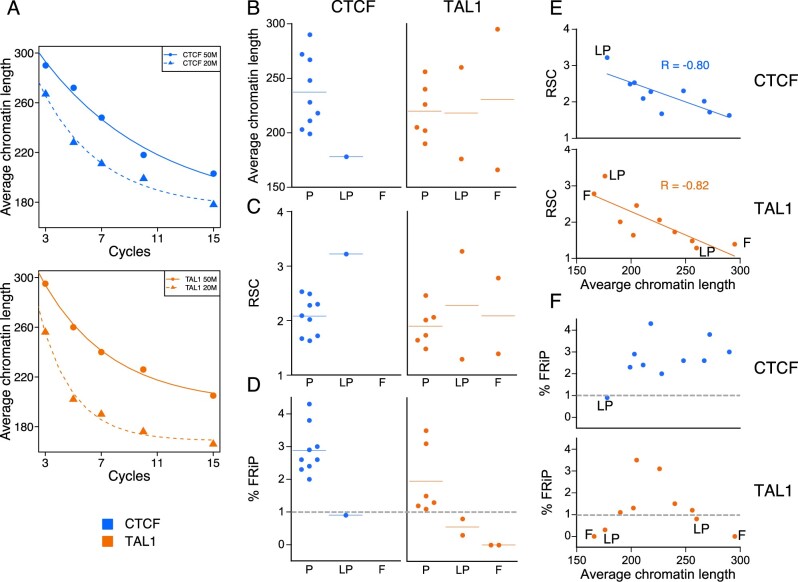
Effect of sonication cycles on chromatin length, and assessment of ChIP-seq success as related to subjective and objective quality metrics. (A) Average chromatin length versus number of sonication cycles for CTCF and TAL1, circles = 50 M cells, triangles = 20 M cells. (B) Average chromatin length classified by subjective assessment, (C) RSC scores classified by subjective assessment. (D) FRiP scores (as percentage) classified by subjective assessment. (E) RSC versus chromatin size for CTCF and TAL1. (F) FRiP (as percentage) versus average chromatin size for CTCF and TAL1. blue, CTCF; orange, TAL1; P, pass; LP, low pass; F, fail. Blue and orange horizontal lines in each category in panels (B) through (D) represent the mean. Gray dotted lines in panels (D) and (F) indicate the commonly used 1% threshold for FRiP.

We found that, as expected, the chromatin size decreased exponentially relative to the numbers of cycles of sonication that were used for generating both the CTCF and TAL1 ChIP-seq datasets (Figure 2A). The number of cycles required to reach a given average chromatin size range depended on the number of cells in the starting sample, with consistently more cycles of sonication needed to break chromatin to a given size when more cells are being processed. One would also expect sonication behavior to vary with different cell types and fixation methods, so in practice, the number of cycles to obtain a particular range of chromatin needs to be determined empirically. A general procedure for determining the optimal number of cycles to obtain a good fragment size range is outlined in Supplementary Figure S1.

We then tested the hypothesized impact of chromatin size on the success rate of ChIP-seq in several ways. First, we categorized the success or failure of each ChIP-seq experiment by inspection of the signal tracks in loci for which the CTCF and TAL1 patterns have been studied extensively by ChIP-seq and genetic experiments (Wilson *et al.* 2010, 2016; Dogan *et al.* 2015; Xiang *et al.* 2020)*.* As illustrated for the *Gfi1b* locus (Figure 1, A–D), datasets with good signal-to-noise ratios and concordance with prior knowledge were classified as “Pass,” those with some peaks present but missing others were classified as “Low pass,” and those with almost no peaks were classified as “Fail” (Figure 1, A–D). These subjective inspections were consistent with the objectively determined numbers of peaks called by MACS in each sample, with the experiments assessed as “Pass” having more peaks (Figure 1, A–D, Supplementary Table S1, Materials and Methods).

We then examined the relationship between the subjective success calls and the input chromatin size, and found that the successful ChIP-seq experiments all came from sheared chromatin whose size was within a fairly defined range (about 190 to 290 bp; Figure 2B). Samples for which the ChIP-seq failed or marginally passed tended to have chromatin sizes outside this range. These results support our hypothesis and indicate that chromatin size may be related to success frequency of ChIP-seq.

We then examined objective quality metrics of each ChIP-seq experiment, starting with the relative strand correlation (RSC) values, which are independent of peak calling (Landt *et al.* 2012). The RSC metric is based on the observation that, in good quality ChIP-seq experiments, mapped reads accumulate on the forward and reverse strands centered around the binding site and separated by a distance that depends on the fragment length distribution. After computing the cross-correlation of read mapping density on each strand as a function of a shifting distance, the RSC value is determined as the ratio of the cross-correlations at the peak inferred to be related to the fragment length and at a peak representing the read length. The ENCODE standards considered RSC values above 0.8 as indicative of a successful ChIP-seq (Landt *et al.* 2012). In our datasets, the samples with ChIP-seq patterns that passed our subjective visual inspection tended to have RSC values within a defined range (between 1.5 and 2.5) while lower quality datasets tended to have RSC values outside of this range (Figure 2C).

We then examined the Fraction of Reads in Peaks (FRiP) scores, which are dependent on peak calling, for each of our datasets. The FRiP score is determined by calculating the fraction of all mapped reads that fall into peak regions identified by a peak-calling algorithm such as MACS (Landt *et al.* 2012). Even in successful ChIP-seq experiments, only a minority of the sequencing reads map to enriched genomic regions that represent occupied sites while the remainder of the reads represent background or noise, and thus, FRiP is considered to be a useful, straightforward metric for the ChIP-seq success. In our study, the FRiP values aligned with our initial assessment of data quality such that all five of our datasets with reduced signal strength had lower FRiP scores ranging from 0 to 0.9%, thus suggesting a sub-par or failed immunoprecipitation (Figure 2D). Conversely, all of our other study datasets had higher FRiP scores (≥1%), consistent with the 1% threshold in the ENCODE guidelines (Landt *et al.* 2012) and indicative of successful immunoprecipitation. By contrast, other quality metrics we tested (normalized strand coefficient, Q-tag) did not consistently distinguish between successful and unsuccessful immunoprecipitation (data not shown).

As a further test of our hypothesis that chromatin size is one determinant of ChIP-seq quality, we examined whether these objective measures of quality were also related to chromatin size. For these ChIP-seq samples, the RSC score was found to be strongly negatively associated with chromatin size (Figure 2E), and the samples at both the low and high extremes of the sizes were failures (F) or low passes (LP). The FRiP scores were also related to input chromatin size, with the scores for CTCF ChIP-seq datasets increasing with fragment length (Figure 2F). The scores for TAL1 ChIP-seq presented a more complex pattern, such that the datasets with low FRiP scores also had the smallest (<180 bp) and largest (>250 bp) average chromatin length (Figure 2F). As with the RSC assessment, the datasets at the extremes of the fragment size distribution tended to have FRiP scores indicative of poor quality. Overall, these analyses implicate the chromatin fragment size distribution (summarized as the mean) as an important determinant of success of a ChIP-seq experiment, and suggested the possibility that chromatin length may have some predictive value in determining ChIP-seq success.

Since FRiP is sensitive to the parameters used for peak calling, and the differences in the number of peaks called for each dataset may affect the FRiP score for the datasets, we then investigated the impact of using one high confidence, reference peak set to compute FRiP scores for the datasets. Use of the same set of peaks across all datasets potentially could provide a more direct comparison of enrichment between datasets, but it also requires counting of reads mapping to some regions not called as peaks in a particular dataset. No ChIP-seq datasets are true gold-standard reference sets, so we chose objectively defined reference sets based on reproducibility. Specifically, we chose to use “optimal” IDR thresholded peak sets from ENCODE as proxies for high confidence peak sets for both CTCF (28,636 peaks) and TAL1 (3,334 peaks) in G1E-ER4+E2 cells. A high percentage of called CTCF peaks (average of 97%) in our datasets overlapped with the high confidence peaks (CTCF overlapped-hc; Supplementary Figure S2A, Table S2), whereas a smaller fraction of called TAL1 peaks in the pass and low-pass datasets overlapped with the high-confidence set, ranging from 23 to 51% (mean of 41%) (TAL1 overlapped-hc; Supplementary Figure S2B, Table S2). We then computed the fraction of reads that are in the overlapped-hc peak sets for CTCF and TAL1 for each of the datasets, generating a variant of the FRiP score (FRiP-hc) (Supplementary Figure S2, C and D). As one might expect, the FRiP-hc scores were lower than the FRiP scores for the higher quality ChIP-seq results, likely reflecting the smaller number of peaks in the overlapped-hc sets, while the lower quality ChIP-seq results showed an increase in FRiP-hc relative to FRiP, perhaps because of the inclusion of reads in peaks not called in those datasets (Supplementary Figure S2, E and F). Importantly, even with this reduced range of values, the FRiP-hc scores are consistently higher for the subjectively assessed “pass” datasets than for the “low-pass” or “failed” sets. Thus, the observation of lower quality or failed datasets having lower FRiP-like scores than higher quality datasets is robust to the choice of peak sets used.

### Motif analysis

Having shown that sonication and the resulting distribution of chromatin fragment sizes are important variables in ChIP-seq experiments, we considered what aspects of ChIP-seq data and their interpretation were sensitive to variation in sonication conditions. As just presented, the signal-to-noise ratio was heavily influenced, leading to lower FRiP scores in the poor-quality datasets. Also, the number of peaks was substantially reduced in low quality and poor datasets. We then investigated the impact of sonication variation on inferences of direct or indirect binding. The TF-bound sites deduced from ChIP-seq experiments are a mix of both direct binding to DNA with binding site motif and indirect binding to another DNA-bound protein, and either or both types of binding could be reduced in the lower quality datasets.

**Figure 3:**
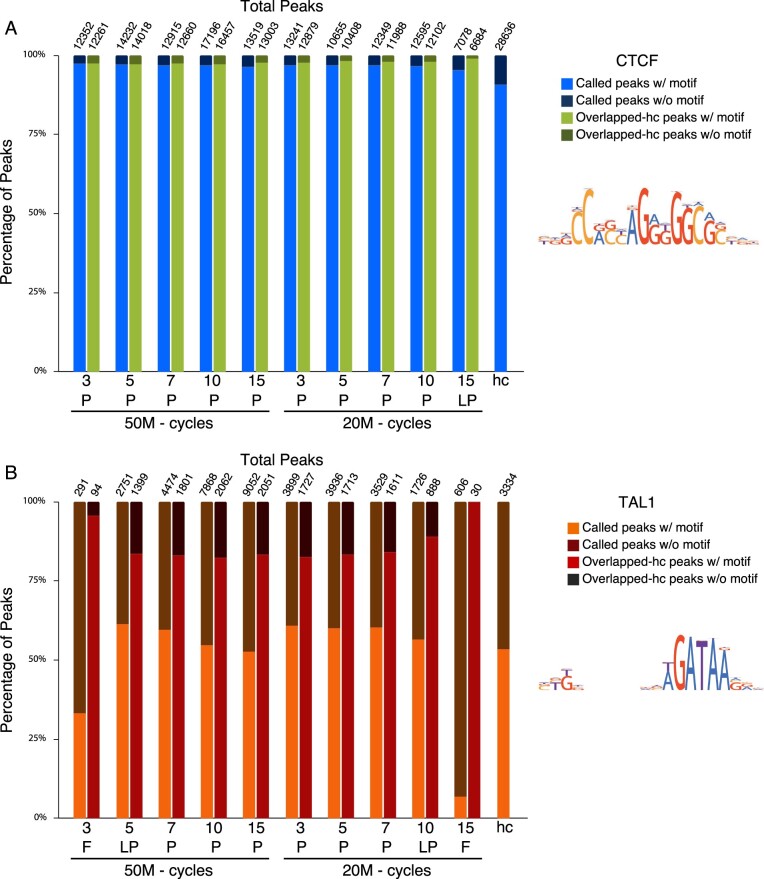
Peaks and overlapped-hc peaks, split by the presence or absence of a significant match to a binding site motif (*P* < 0.0001). High confidence peaks (hc) included for reference. Consensus binding sites for CTCF and TAL1 motifs maintained by HOCOMOCO (v11) shown on the right. (A) CTCF. (B) TAL1. Blue, CTCF; green, CTCF overlapped-hc peaks, orange, TAL1 peaks; brown, TAL1 overlapped-hc peaks.

We assessed the contributions of direct and indirect binding to the peak sets in our experiments by using the presence of a significant match to a binding site motif in the peak region as the indicator of direct binding. Using the program FIMO (5.2.0) (RRID: SCR_001783), we scanned the DNA sequences of the called peaks in our datasets for significant (*P *<* *0.0001) matches to the CTCF and TAL1 binding site motifs recorded in HOCOMOCO (v11) (RRID: SCR_005409) (Supplementary Table S2). Approximately 97% of called CTCF peaks in each dataset contained the consensus binding site motif, with little variation among the datasets despite differences in the total numbers of peaks and FRiP scores (Figure 3A). Similarly, CTCF peaks that overlapped the high confidence set also had a very high frequency of motif presence (mean of 98%. range = 97–99%, *P *<* *0.0001, Figure 3A). Thus, we infer that almost all the CTCF peaks reflect direct binding, even for the low-pass dataset. Peaks lost in the lower quality dataset predominantly are inferred to result from direct binding.

In contrast, slightly over half of TAL1 peaks (mean of 58%, range = 53 − 61%; *P *<* *0.0001) contained a significant match to the binding site motif in all the successful (pass and low pass) and high confidence TAL1 datasets (Figure 3B). This lower proportion of direct binding for TAL1 (compared to CTCF) is expected for this TF, which is known to bind in association with other proteins at many locations (Wu *et al.* 2014). Importantly, the failed TAL1 ChIP-seq experiments showed little to no motif enrichment, indicating that the peaks called in these low-quality datasets reflect indirect binding or false positives. When the motif analysis was confined to the peaks that overlapped the high confidence TAL1 peaks, a larger proportion contained a motif match (mean of 87%, range = 82–100%; *P *<* *0.0001; Figure 3B), suggesting that the subset of called peaks overlapping high confidence peaks was enriched for direct binding. Despite the rarity of a motif match in the small numbers of peaks in the failed TAL1 ChIP-seq experiments, the very small number of peaks in those sets that overlap the high confidence set actually do contain a match to the binding site motif. These results indicate that for TAL1 ChIP-seq, peaks inferred to result from both direct and indirect binding were lost in the lower quality and failed experiments, but a small number of direct binding peaks were retained.

**Figure 4:**
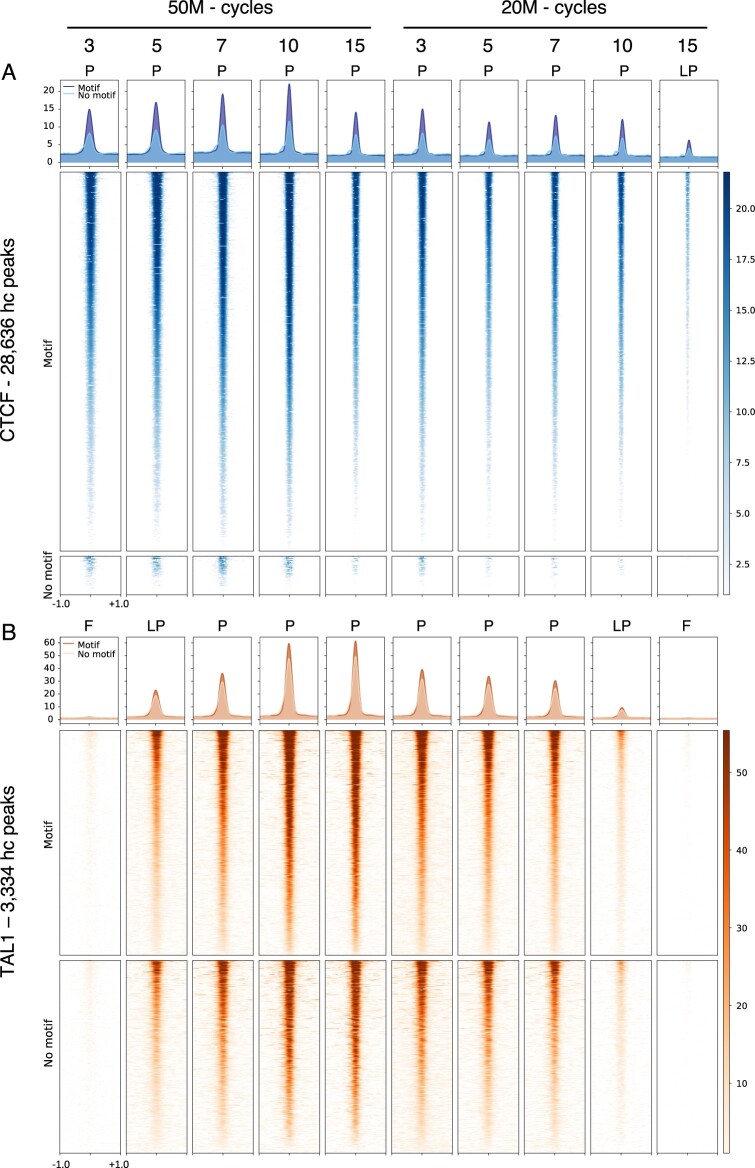
Metapeak analysis on the high confidence set of peaks for (A) CTCF, and (B) TAL1 datasets. Each peak was classified by the presence or absence of a significant match to the binding site motif. Each row represents a genomic region (centered on a peak and then extended 1,000 bp in each direction) with the signal strength (normalized as the Z-score for mapped reads) indicated by the color intensity, with darker colors for higher scores. Aggregated signal intensity across all DNA intervals are plotted in the top graphs for peaks containing a motif match (darker color) or not (lighter color). Peaks with a significant CTCF motif (*P *<* *0.0001) = 25984. Peaks without a CTCF motif = 2651. Peaks with a significant TAL1 motif (*P *<* *0.0001) = 1783. Peaks without a TAL1 motif = 1550. blue, CTCF; orange, TAL1.

We then performed a metapeak analysis to examine at higher granularity how the ChIP-seq read distribution at peak sites changed as a function of sonication and chromatin size. For consistency, we computed the numbers of mapped reads across the DNA intervals containing high confidence peaks for all experiments, regardless of whether a peak was called in each experiment. In the CTCF datasets, the motif-containing peaks presented a stronger signal compared to those that did not contain a significant motif match, and the signal in both types of peaks was reduced in the low pass dataset (Figure 4A). In contrast, the signal intensities and positional distributions are similar for the TAL1 peaks in both the motif-containing class and the motif-lacking class (Figure 4B), again leading to the inference that TAL1 binds directly or indirectly at roughly equivalent frequencies. The signal intensities were reduced for both classes of TAL1 binding in the low-pass datasets, and they were almost undetectable for both classes in the failed datasets.

Both types of analyses indicate that sonication conditions that produced lower quality datasets reduced the ability to detect both direct and indirect binding for both TFs. The failed experiments detected few peaks, but even in these cases, a low-level accumulation of reads was detected at the positions of high confidence peaks.

### Retrospective analysis

**Figure 5:**
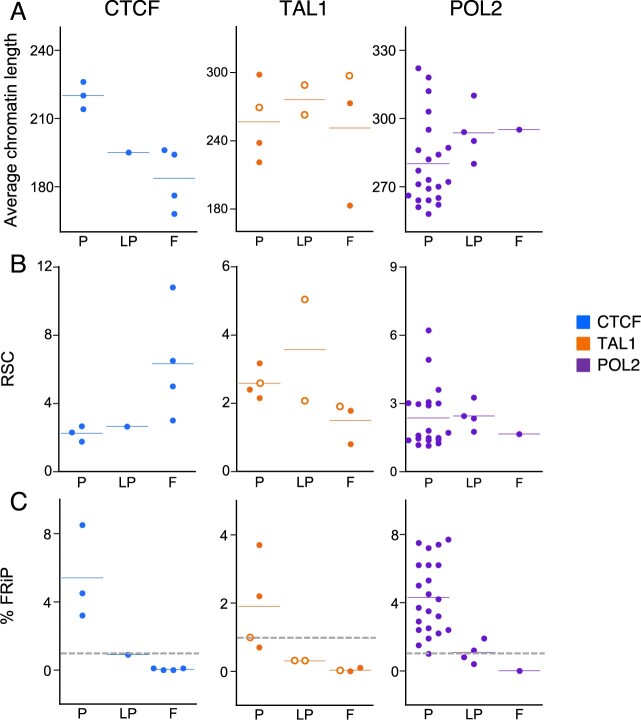
Retrospective datasets separated by subjective assessment. (A) Average chromatin length. (B) RSC. (C) FRiP (as percentage). blue, CTCF; orange, TAL1; purple, POL2; P, pass; LP, low pass; F, fail; open circles, different fixative than all other TAL1 datasets (new and retrospective) described in this study. Blue and orange horizontal lines in each category represent the mean. Gray dotted lines in (C) indicate the commonly used 1% threshold for FRiP.

To examine whether the relationship between average fragment size and success rate of ChIP-seq held for other samples, we did a retrospective analysis of 17 additional ChIP-seq datasets (eight for CTCF and nine for TAL1) generated in our laboratory. These ChIP-seq experiments were conducted over a variety of conditions, including different fixation methods that confounded our assessment of quality, and they provided an opportunity to determine whether a robust relationship between chromatin size and quality could be detected. After assessing their quality by subjective inspection, we observed, as before, that very low average chromatin length was associated with failure or low quality of the experiment (Figure 5A). Successful ChIP-seq experiments showed values around 2 for the ENCODE quality metric RSC and a FRiP score above 1% (Figure 5, B and C, Supplementary Table S1). While no consistent trend was observed for RSC scores in low quality or failed datasets, all such datasets had very low FRiP scores. These retrospective analyses support our hypothesis that chromatin length distributions are important variables in the success of ChIP-seq. However, we also found that some of the retrospective TAL1 datasets with larger average chromatin size ranges were successful, particularly those prepared with different fixatives.

To determine whether the effect of chromatin size generalizes among different factors and even between different laboratories, we included a series of published and unpublished ChIP-seq experiments on a third protein, POL2, in our retrospective analysis. These experiments examined the pattern of POL2 occupancy over a time-course after release of mitotically arrested G1E-ER4+E2 cells (Hsiung *et al.* 2016). The mitotic cells had very low levels of transcription and POL2 occupancy, but those levels increased dramatically after release to G1 phase. Therefore, we included only the POL2 datasets at least 60 minutes after release to focus on the period after transcription was underway, and we only compared replicates within each time point after release for our subjective assessment of dataset quality (Supplementary Table S1). We could not examine the impact of very small average chromatin lengths in these POL2 ChIP-seq datasets because all were >260 bp, but we did find that the lower quality and failed ChIP-seq experiments all had average chromatin lengths of ≥290 bp (Figure 5A). No consistent trend was observed for RSC scores, but the lower quality and failed POL2 experiments had lower FRiP scores than successful POL2 experiments (Figure 5, B and C). Thus, this retrospective analysis of experiments for all three proteins further supports the finding that average fragment length distribution (sonication) impacts the quality and sensitivity of ChIP-seq.

**Figure 6:**
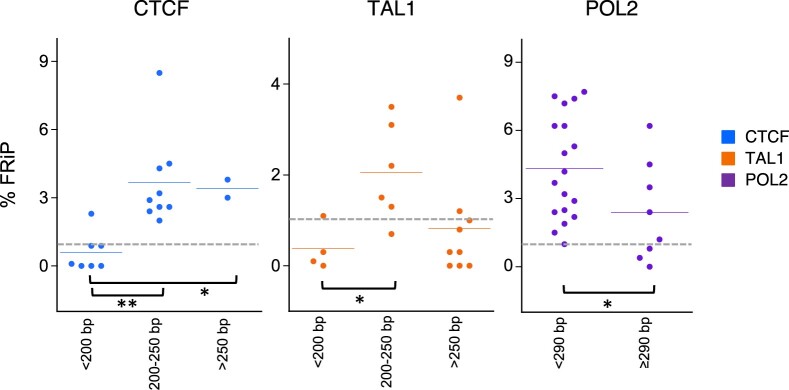
Relationship between chromatin size and FRiP score (as percentage) for study and retrospective datasets. blue, CTCF; orange, TAL1; purple, POL2. Blue and orange horizontal lines in each category represent the mean. *P <* *0.05, **P <* *0.005. Gray dotted lines indicate the commonly used 1% threshold for FRiP.

To further test our hypothesis that ChIP-seq experimental success was dependent on the input chromatin size, we conducted a meta-analysis of the combined initial and retrospective datasets using the quality metric FRiP score rather than pass-fail categorization. These combined datasets from a total of 62 experiments were binned by the chromatin length, specifically <200, 200–250, and >250 bp for CTCF and TAL1, and <290 and ≥290 bp for POL2 (Figure 6). Our hypothesis predicts that the FRiP scores should be higher for experiments with chromatin sizes in specific ranges for each factor, and this was observed in the results. For both CTCF and TAL1, most samples of chromatin size <200 bp have a low FRiP score indicative of failure, while experiments with samples of chromatin size between 200 and 250 bp have FRiP scores indicative of success. The differences between the scores for these two size ranges were significant for both CTCF (*P *<* *0.005, Student's *t*-test) and TAL1 (*P *<* *0.05, Student's *t*-test). Consistent with the previous analyses, CTCF ChIP-seq was successful on samples with an average fragment size larger than 250 bp. Most of the TAL1 ChIP-seq experiments on samples in this larger size range had low FRiP scores, but some were successful, showing that the impact of larger chromatin fragment sizes on success of TAL1 ChIP-seq was not completely consistent. These results support our hypothesis and show that, under the conditions tested, sonication products between 200 and 250 bp yield the highest frequency of acceptable FRiP scores for successful CTCF and TAL1 ChIP-seq experiments. For the retrospective POL2 datasets, samples with a mean average chromatin length <290 bp had high FRiP score indicative of success, while experiments with samples of chromatin size ≥290 bp tended to have lower FRiP scores indicative of either lower quality or failed ChIP-seq experiments (*P *<* *0.005, Student's *t*-test). These latter data, despite the fact that they were generated in another lab with a different sonicator, provide an additional example of the impact of fragment length distribution (sonication) on the success rate of ChIP-seq, thus further supporting our hypothesis.

Finally, several retrospective datasets provided an opportunity to examine whether using a matched versus a nonmatched input control for peak calling with MACS impacted the number of called peaks or the FRiP quality scores. We do not typically sequence unenriched chromatin from the same cell line multiple times, and instead use a composite, nonmatched input file generated from several different sequenced G1E-ER4+E2 libraries to call peaks with MACS (Supplementary Table S3) for the majority of ChIP-seq experiments that use G1E-ER4+E2 cells. By re-analyzing four TAL1 retrospective datasets using their matched input sequence data, we found that using a matched input control had little effect on peak numbers and the FRiP scores were unchanged (Supplementary Figure S3).

### Low input ChIP-seq

**Figure 7:**
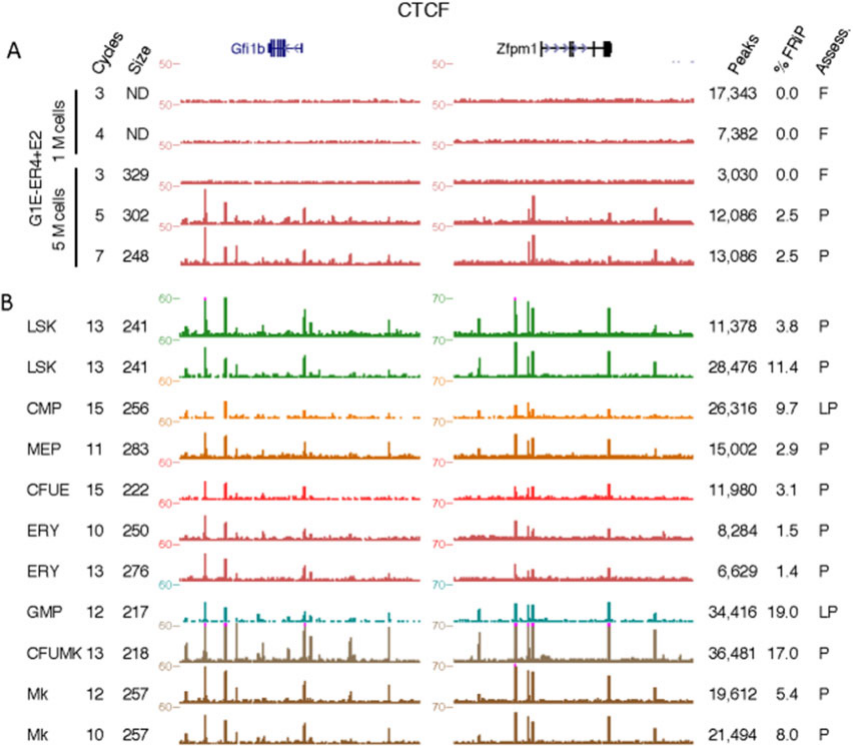
CTCF occupied sites at the *Gfi1b* (chr2: 28,575,695—28,666,063) and *Zfpm1* (chr8: 122,210,058—122,399,621) loci. Shown to the left of the genome browser tracks are the number of sonication cycles and the mean size of the unenriched chromatin. Shown to the right of the genome browser tracks are peaks, FRiP score (as percentage), and subjective assessment of each dataset. (A) G1E-ER4+E2 cells, and (B) Hematopoietic cell types LSK, Lin^-^Sca1^+^Kit^+^; CMP, common myeloid progenitor cells; MEP, megakaryocyte erythrocyte progenitor cells; CMP, common myeloid progenitor cells; CFUE, colony-forming units erythroid; ERY, erythroblasts; GMP, granulocyte monocyte progenitor cells; CFUMK, colony-forming units megakaryocyte; MK, megakaryocytes. P, pass; L, low pass; F, fail; ND, not detectable.

Obtaining reliable ChIP-seq results on TFs or other epigenetic features in cell types that can be purified only in small quantities would facilitate many studies, such as mechanisms regulating gene expression during cell differentiation. While some success has been reported for ChIP-seq in low cell input samples (Adli *et al.* 2010; Lara-Astiaso *et al.* 2014), these approaches have been applied primarily to modified histones, which are often present in greater abundance than TFs. We wanted to determine whether careful control of chromatin shearing would facilitate low input ChIP-seq using standard methods. A pilot CTCF ChIP-seq experiment varying sonication cycles in 1 and 5 M G1E-ER4+E2 cells showed that successful results could be obtained with 5 M cells with appropriate sonication (Figure 7A, Supplementary Table S1). No signal was observed in any of the 1 M cell samples or the 5 M cell sample with an average chromatin length of 329 bp. However, we observed good quality signal tracks with FRiP scores ≥1.0% in the 5 M cell datasets with average chromatin lengths of 302 and 248 bp.

We then applied our findings on low cell input to produce a set of CTCF ChIP-seq datasets in hematopoietic cell populations purified by sorting mouse BM cells (Figure 7B, Supplementary Table S1, Materials and Methods). These cell populations were LSK (Lin^−^Sca1^+^Kit^+^, which includes hematopoietic stem cells or HSC), several multilineage progenitor cells (common myeloid progenitor cells or CMP, granulocyte monocyte progenitor cells or GMP, megakaryocyte erythrocyte progenitor cells or MEP), and committed cells of two major blood cell lineages at different stages of maturity, specifically colony-forming units erythroid (CFUE) and erythroblasts (ERY) and colony-forming units megakaryocyte (CFUMK), and megakaryocytes (MK). The progenitor cells are very rare in the BM, and it is challenging to obtain enough cells for a single ChIP-seq determination. For example, the isolation of 4.85 M MEP cells required isolation of BM from 200 mice and a total of 10 flow cytometry sorts (Supplementary Table S4). The progenitor cells were isolated and fixed in small batches (<1 M cells, Materials and Methods) over the course of several months, and they were frozen at −80°C until enough cells were amassed to attempt a CTCF ChIP-seq experiment. Isolation procedures of this magnitude involve a significant time investment, and thus it is critical on the part of the experimentalist to do everything possible to increase the probability of ChIP-seq success.

As before, we used the Diagenode Biorupter Plus 300 to shear chromatin from the fixed hematopoietic cells, assayed the extent of shearing using the Agilent Bioanalzyer 2100, and then subjected the chromatin to immunoprecipitation and sequencing using antiserum against CTCF (Figure 7B). We first inspected each of the signal tracks in all 11 datasets in eight different cell types across several loci, including the *Gfi1b* and *Zfpm1* loci. We observed peaks in all 11 datasets across eight different cell types examined, though there were notable differences in the signal-to-noise ratio between samples. Based on this initial subjective visual inspection, two of the 11 datasets were deemed as “Low pass” while the remaining datasets were classified as “Pass.” Examination of the objective metrics for these datasets revealed a large number of peaks and, in some cases, very high FRiP scores with all of the datasets having a FRiP score of at least ≥1%.

In summary, by rigorous attention to sonication conditions, we were able to obtain good quality CTCF binding profiles across a panel of cells differentiating from rare, multilineage progenitor cells to lineage-specific, maturing blood cells.

## Discussion

ChIP-seq has been used extensively across a broad spectrum of species, tissues, and cell types to interrogate the locations of TFs occupying specific sites in chromatin or mapping the profile of histone modifications in chromatin (Wold and Myers 2008; Rivera and Ren 2013). This powerful technique has moved studies of gene regulation to a global (genome-wide) scale, and the data produced by this method form the foundation for many efforts to develop coherent, integrated models for gene regulation (Ching *et al.* 2018; Zhou *et al.* 2019; Xiang *et al.* 2020). However, the technique is not uniformly successful for all samples, and even when apparently successful, the resulting ChIP-seq datasets vary widely in quality (Marinov *et al.* 2014; Devailly *et al.* 2015). A major factor impacting success of the ChIP-seq is the antibody directed against the chromatin-associated factor, *i.e.*, TF or histone modification (Landt *et al.* 2012). Effective antibodies not only must be highly specific, but they also must recognize epitopes that may not be sufficiently exposed in the fixed chromatin. These requirements are difficult to evaluate prior to performing the ChIP-seq experiment, since many antibodies that are effective for other applications, such as Western blots, are not effective in ChIP-seq (Egelhofer *et al.* 2011). Thus, the only assay for effectiveness of an antibody is to actually do the ChIP-seq experiment, which requires expenditure of resources and time, and of course it is frustrating when the experiment fails. Such failures are frequent for commercially available antibodies (Egelhofer *et al.* 2011; Savic *et al.* 2015). Sophisticated tagging approaches have been developed to enable the use of antibodies known to be effective in a context where the level of antigen is close to the natural level (Savic *et al.* 2015). These approaches help to generate more uniform results for TFs expressed in cell lines or organisms amenable to the targeted genetic engineering required for tagging.

Other factors also contribute to the success of a ChIP-seq experiment, some of which are directly under the control of the experimentalist. One illustration of high-technical variation arises when a particular lot of a commercial antibody preparation is used successfully in ChIP-seq for one biosample, but then a subsequent experiment fails, even though the same lot of antibody was used in another preparation of that same biosample. The experiments described in this study were developed to better understand the technical contributors to success of a ChIP-seq experiment beyond the well-known issues with antibody quality.

We discovered that the size distribution of the sonicated chromatin was an important factor in the ChIP-seq procedure, with more frequent success observed for chromatin with an average size in a range of approximately 200 to 250 bp. We conclude that monitoring the level of chromatin sonication is one way to improve ChIP-seq quality and reproducibility. Indeed, we would argue that in situations with failure and success for the same antibody in the same biosample, it would be wise to re-evaluate the unenriched chromatin length for these samples. In such instances, it is critical to measure the size distribution of the actual chromatin to be used in a ChIP-seq experiment.

The best sonication conditions should be determined empirically for the number and types of cells, and ideally matching any treatments applied to those cells that could cause changes in cell morphology. While some parameters for sonication may be expected to be predictable, the many factors impacting the final chromatin size distribution mean that an empirical approach is needed. For example, as expected, we observed that samples with larger numbers of cells required more sonication cycles than those with fewer cells to achieve a given size range. However, it is should be noted that very low numbers of cells may actually require more sonication cycles than larger numbers of cells because the incidence of a sound wave interacting with the chromatin decreases as the overall chromatin size decreases, relative to the wavelength of the insonifying sound (Espana *et al.* 2014). Thus, additional time may be required to have sufficient interaction between the sound wave and the chromatin. While not specifically addressed in the current presentation, it is reasonable to expect the amount of required sonication to vary by tissue and cell type. To facilitate these empirical determinations, we provide a protocol for determining chromatin size distribution in the desired range in Supplementary Figure S1.

We demonstrated an effect of chromatin size distribution on success of ChIP-seq experiments for three TF targets. While the experiments for both tended to fail at small chromatin sizes for CTCF and TAL1, the effects of larger chromatin sizes differed, with the CTCF ChIP-seq experiments being less sensitive to large sizes of chromatin, compared to TAL1 ChIP-seq. One hypothesis to explain the differences in effects of larger chromatin sizes is a difference in the exposure of the antigens. Specifically, one can conjecture that TAL1 antigens are more sequestered in longer chromatin fragments, whereas the CTCF antigens may be more exposed in those longer fragments. Such accessibility may also be influenced by fixation conditions which have been demonstrated to affect chromatin shearing dynamics (Khoja *et al.* 2019). Relatedly, several of the successful retrospective TAL1 experiments with higher average chromatin lengths were cross-linked with a different fixative than all of the other TAL1 ChIP-seq experiments reported in this study (Supplementary Table S1), and thus it is possible that these differences contributed to accessibility of the epitope and its effect on ChIP-seq quality. However, it is also possible that the effect of larger average chromatin lengths on TAL1 ChIP-seq success is less robust than that of smaller average chromatin lengths. Indeed, we emphasize that sonication and average chromatin length is only one of several variables that affect ChIP-seq quality and sensitivity. Furthermore, we may expect the details of the relationship between chromatin fragment size and ChIP-seq success to vary among different TF targets and fixation conditions, but working within a range with frequent success (200–250 bp for average fragment sizes) is a good starting point for most experiments involving sequence-specific TFs. By comparison, targets that display a high level of enrichment, such as POL2, are likely to be successful under a broader range of acceptable chromatin sizes simply because of the abundance of available epitopes.

In agreement with previous work (Landt *et al.* 2012), we found that FRiP scores of at least 1% to be strongly associated with successful ChIP-seq experiments targeting TFs. It is important to note, however, that the suggested 1% FRiP guideline is most applicable for sequence-specific TFs that have thousands to tens of thousands of occupied sites in large mammalian genomes, and that successful ChIP-seq experiments with TFs that have only a small number of occupied sites would be expected to have a FRiP of <1%. Indeed, successful ChIP-seq experiments for ZNF410, a pentadactyl DNA-binding protein that is expressed in human erythroid cells and directly activates only a single gene, typically have FRiP scores of <0.2% (Lan *et al.* 2021). By contrast, for TFs with very large numbers of occupied sites (*e.g.*, RAD21) and/or those that display a high level of enrichment at binding locations, may actually have FRiP scores >1%, despite a lower quality or failed ChIP-seq experiment (Landt *et al.* 2012). Thus, while we used FRiP to compare ChIP-seq experiments obtained with the same antibody, we emphasize that FRiP scores are not comparable between different antibody targets (CTCF vs TAL1 vs POL2). We also noted some experimental results with very high FRiP scores, *e.g.*, 17–19%. Such very high scores do not necessarily indicate an exquisite dataset. For instance, in a dataset with a low signal-to-noise ratio, a peak-calling algorithm may call an excessive number of false positive “peaks.” Those overcalled peaks are included when counting the number of reads assigned to peaks, and thus, the FRiP score can be artificially inflated.

In addition to improving the success rate for conventional ChIP-seq experiments, careful control of chromatin shearing may help facilitate ChIP-seq experiments on low numbers of input cells. We showed that, with rigorous attention to sonication conditions, we were able to obtain good quality CTCF binding profiles across a series of cells differentiating from multilineage progenitor cells to lineage-specific, maturing blood cells. This series includes rare progenitor cells that have been difficult to interrogate for binding profiles of specific TFs. These maps of binding by CTCF across progenitor and lineage-specific cells should be useful for multiple studies of the roles of this important architectural protein during blood cell differentiation.
